# Relationship Quality and Depressive Symptoms Among Very Old Parents and Their Older Children in South Korea

**DOI:** 10.1093/geroni/igaa057.1119

**Published:** 2020-12-16

**Authors:** Gyounghae Han, Joohong Min, Joohyun Kim, Kyungmin Kim, Kathrin Boerner

**Affiliations:** 1 Seoul National University, Seoul, Republic of Korea; 2 Jeju National University, Jeju, Republic of Korea; 3 University of Massachusetts Boston, Boston, Massachusetts, United States

## Abstract

Research has consistently reported the association between intergenerational relationship quality and mental health outcomes in later life. However, few studies have examined the link among very old parents and their older children, and even fewer studies investigated whether the relationship quality matters similarly to parents and children. Employing a dyadic approach, this study examined how one’s own and partner’s perceptions of relationship quality (i.e., support and conflict) are associated with depressive symptoms among very old parents and their children. Data from 105 dyads of parents (age 81-97; M = 87.92, SD = 2.80) and their children (age 65-72; M = 65.87, SD = 1.23) in South Korea were used. Results showed that parents tended to report significantly higher levels of intergenerational support and lower levels of intergenerational conflict, compared to their children. Regarding the actor effects of relationship quality, one’s own perceptions of intergenerational conflict were positively associated with depressive symptoms for both parents (β = 0.26, p < .01) and children (β = 0.37, p < .001), whereas intergenerational support was not significant. In terms of the partner effect, intergenerational support (reported by parents) was negatively related to depressive symptoms only for children (β = -0.21, p < .01), but the partner effect of conflict was not significant. The findings highlight the centrality of perceptions of intergenerational relationship in understanding well-being in later life. Further, children’s depressive symptoms were susceptible to how their parents view the relationship. Findings were discussed in the context of Intergenerational Stake Theory.

